# *Nucks1* synergizes with *Trp53* to promote radiation lymphomagenesis in mice

**DOI:** 10.18632/oncotarget.11297

**Published:** 2016-08-16

**Authors:** Yangbo Yue, Stanley G. Leung, Yueyong Liu, Yurong Huang, Kirsten Grundt, Anne-Carine Østvold, Kuang-Yu Jen, David Schild, Jian-Hua Mao, Claudia Wiese

**Affiliations:** ^1^ Department of Organismal Systems and Bioresilience, Biological Systems and Engineering Division, Lawrence Berkeley National Laboratory, Berkeley, CA 94720, USA; ^2^ Department of Molecular Medicine, Institute of Basic Medical Science, University of Oslo, 0317 Oslo, Norway; ^3^ Department of Pathology and Laboratory Medicine, University of California, Davis, CA 95817, USA; ^4^ Department of Environmental and Radiological Health Sciences, Colorado State University, Fort Collins, CO 80523, USA; ^5^ Present address: Department of Dermatology, University of Texas, Southwestern Medical Center, Dallas, TX 75390, USA

**Keywords:** NUCKS1, double-strand break repair, ionizing radiation, thymic lymphoma, V(D)J recombination

## Abstract

NUCKS1 is a 27 kD vertebrate-specific protein, with a role in the DNA damage response. Here, we show that after 4 Gy total-body X-irradiation, *Trp53*+/− *Nucks1*+/− mice more rapidly developed tumors, particularly thymic lymphoma (TL), than *Trp53*+/− mice. TLs in both cohorts showed loss of heterozygosity (LOH) of the *Trp53*+ allele in essentially all cases. In contrast, LOH of the *Nucks1*+ allele was rare. *Nucks1* expression correlated well with *Nucks1* gene dosage in normal thymi, but was increased in the majority of TLs from *Trp53*+/− *Nucks1*+/− mice, suggesting that elevated *Nucks1* message may be associated with progression towards malignancy *in vivo*. *Trp53*+/− *Nucks1*+/− mice frequently succumbed to CD4- CD8- TLs harboring translocations involving *Igh* but not *Tcra/d*, indicating TLs in *Trp53*+/− *Nucks1*+/− mice mostly originated prior to the double positive stage and at earlier lineage than TLs in *Trp53+/*- mice. Monoclonal rearrangements at *Tcrb* were more prevalent in TLs from *Trp53*+/− *Nucks1*+/− mice, as was infiltration of primary TL cells to distant organs (liver, kidney and spleen). We propose that, in the context of *Trp53* deficiency, wild type levels of *Nucks1* are required to suppress radiation-induced TL, likely through the role of the NUCKS1 protein in the DNA damage response.

## INTRODUCTION

Ionizing radiation (IR) is an environmental carcinogen, and exposure to IR is associated with negative effects on health, such as reduced hematopoietic cell function and an elevated risk for cancer. These malignancies are considered to also result from the direct induction of mutations due to insufficient or imprecise repair of DNA damage after IR. IR induces a variety of DNA lesions, of which DNA double-strand breaks (DSBs) are considered to be the most detrimental [1]. To sense and repair DSBs, cells have evolved numerous highly efficient repair pathways, and the two main pathways for DSB repair in eukaryotes are classical non-homologous end joining (NHEJ) and homologous recombination (HR). Defects in either DNA repair pathway can cause genome instability and tumorigenesis [2, 3].

In addition to choosing the right DSB repair pathway, the capacity of cells to sense DNA damage and to signal to downstream effectors in the DNA damage response (DDR) network is crucial for genome stability and cancer avoidance. For example, when the Ataxia Telangiectasia Mutated Serine/Threonine-Protein Kinase (ATM), one of the key components of the DDR, is mutated, patients develop Ataxia-Telangiectasia (A-T), an autosomal recessive syndrome characterized by progressive neurodegeneration, radiosensitivity, immune dysfunction, cell cycle checkpoint defects and an increased predisposition to cancer [4]. ATM is one of the six members of the phosphoinositide 3-kinase-related protein kinase (PIKK) family that includes other DDR sensors such as AT and Rad3-related protein kinase (ATR) and DNA-dependent protein kinase catalytic subunit (DNA-PKcs).

More than 1,300 proteins are phosphorylated in response to DNA damage, as shown by the results from several studies using quantitative proteomics [5–9]. One such protein is Nuclear Casein Kinase and Cyclin-dependent Kinases Substrate 1 (NUCKS1), a nuclear and highly phosphorylated protein [10–12], which also is acetylated, methylated, ubiquitylated and formylated [13] (http://www.phosphosite.org/). Specifically, in phosphoproteomic screens, NUCKS1 Ser14 was identified as a substrate of either ATM or ATR after IR [5], and Ser54 and Ser181 were identified as ATM-dependent phosphorylation sites after neocarzinostatin [6]. In addition, our own results show that the DNA damage-induced phosphorylation of NUCKS1 at Ser54 is ATM-dependent and that it occurs in HeLa cells both after exposure to IR and after mitomycin C [14]. Collectively, the results from phosphoproteomic screens [5–9] and of our previous investigation [14] show that the NUCKS1 protein is an important new player in the DDR, although its precise functions still remain to be elucidated.

Albeit there are little functional cancer-related data on NUCKS1, several studies suggest that there are some links, particularly between *NUCKS1*/NUCKS1 expression and breast cancer [13, 15–17]. NUCKS1 also was identified as a colorectal cancer prognostic marker [18], as a biomarker for recurrence-free survival in cervical squamous cell carcinoma [19], as a risk factor for poor prognosis and recurrence in endometrial cancer [20], as an immunodiagnostic marker in hepatocellular carcinoma [21], and as aberrantly low expressed in adult T-cell leukemia-lymphoma [22] and in childhood acute lymphoblastic leukemia [23]. However, how expression of *NUCKS1*/NUCKS1 is linked to initiation and/or progression towards malignancy is currently unknown.

Using gene-specific knockdown of *NUCKS1* in human cells, we have shown that NUCKS1 is a chromatin-associated protein with a novel role in the DDR and in HR, a DNA repair pathway critical for tumor suppression [14]. However, whether functional loss of NUCKS1 in mice would lead to an increased susceptibility to cancer had not been explored. Here we show that *Trp53*+/− *Nucks1*+/− mice more rapidly developed tumors than *Trp53*+/− mice after exposure to 4 Gy total-body X-irradiation (TBI). Notably, in *Trp53*+/− *Nucks1*+/− mice IR-induced thymic lymphomas (TLs) were more prevalent and arose at earlier lineage than in *Trp53*+/− mice, frequently with concomitant with an upregulated expression of *Nucks1* transcript. We propose that, in the context of *Trp53* deficiency, wild type levels of murine NUCKS1 are required to suppress radiation carcinogenesis, in line with an important role for NUCKS1 in the DDR.

## RESULTS

### *Nucks1* shows haploinsufficiency for suppressing radiation carcinogenesis in mice

We tested for the consequences of total-body X-irradiation (TBI) of mice with partially inactivated *Nucks1*. We chose to do these experiments in the context of *Trp53* heterozygosity to avoid *Trp53* checkpoint activation in response to partial *Nucks1* deficiency and/or IR exposure [24, 25]. To obtain *Trp53*+/− and *Trp53+/−Nucks1+/−* F2 littermates, a founder mouse heterozygous for *Nucks1* (derived from strain: *Nucks1^Gt(XG374)Byg^* (BayGenomics)) and in a mixed genetic background, was backcrossed to mice heterozygous for *Trp53* (strain: 129S1/SvImJ-*Trp53^tm1Tyj^*/J (Jackson Laboratory)). F1 mice on average were 25% C57BL/6, 62.5% 129S1/SvImJ and 12.5% CBA in genetic background. F1 mice heterozygous for both *Nucks1* and *Trp53* were further backcrossed to wild type 129S1/SvImJ mice to generate F2 mice that on average were 81.25% 129S1/SvImJ, 12.5% C57BL/6 and 6.25% CBA in genetic background (Supplementary Information; Supplementary Figure S1). F2 littermates were used for the experiments described below.

**Figure 1:**
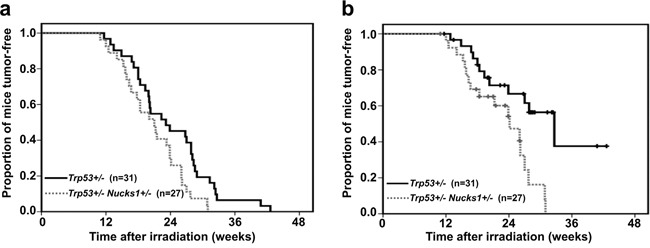
*Nucks1* deficiency promotes radiation carcinogenesis in mice **a.** Kaplan-Meier survival curves for tumor-free survival of *Trp53*+/− *Nucks1*+/− and *Trp53*+/− mice after 4 Gy TBI (*P*=0.018; Log-Rank test). **b.** Kaplan-Meier survival curves for thymic lymphoma (TL)-free survival of *Trp53*+/− *Nucks1*+/− and *Trp53*+/− mice after 4 Gy TBI (*P*=0.004; Log-Rank test).

After 4 Gy TBI, all F2 *Trp53*+/− mice died within 45 weeks (Figure 1a). Tumor spectrum analysis revealed that *Trp53*+/− mice frequently developed TL (38.7%; Table 1). *Trp53*+/− mice also developed sarcoma and splenic lymphoma (Table 1). Tumor latency in F2 *Trp53*+/− *Nucks1*+/− mice was significantly reduced (*P*=0.018; Log-Rank test), and most mice died within 31 weeks (Figure 1a). The median survival times for *Trp53*+/− and *Trp53*+/− *Nucks1*+/− mice were 193 and 175 days, respectively. Tumor spectrum analysis revealed that the fraction of mice succumbing to TL was significantly higher in *Trp53*+/− *Nucks1*+/− mice (66.7%; Table 1; *P*=0.0398; Fisher's exact test). *Trp53*+/− *Nucks1*+/− mice also developed primary lung epithelial tumors that were not observed in *Trp53*+/− single heterozygous mice (Table 1; Supplementary Figure S2). When Kaplan-Meier survival curves for both cohorts were confined to mice with TL only (Figure 1b), a significant difference for TL-free survival between both cohorts was detected (*P*=0.004; Log-Rank test). Median survival times for succumbing to TL were 272 and 201 days for *Trp53*+/− and *Trp53*+/− *Nucks1*+/− mice, respectively.

**Table 1:** Tumor types and frequencies in *Trp53+/−* and *Trp53+/− Nucks1+/−* mice

Tumor type^1^	Tumor frequency (%)	
	*Trp53*+/−	*Trp53*+/− *Nucks1*+/−
Thymic lymphoma	38.7	66.7
Splenic lymphoma	12.9	7.4
Sarcoma	16.7	22.2
Lung tumor^2^	-	11.1
Other tumors	16.7	14.8
Paralysis^3^	22.6	11.1

1 Only primary tumors, as examined by H&E or immunohistochemistry.

2 Epithelial-origin lung tumors were found in **Trp53**+/− **Nucks1**+/− mice only.

3 Mice were characterized as succumbing to paralysis when their limbs, usually their hind limbs, lost motility followed by severe weight loss.

**Figure 2:**
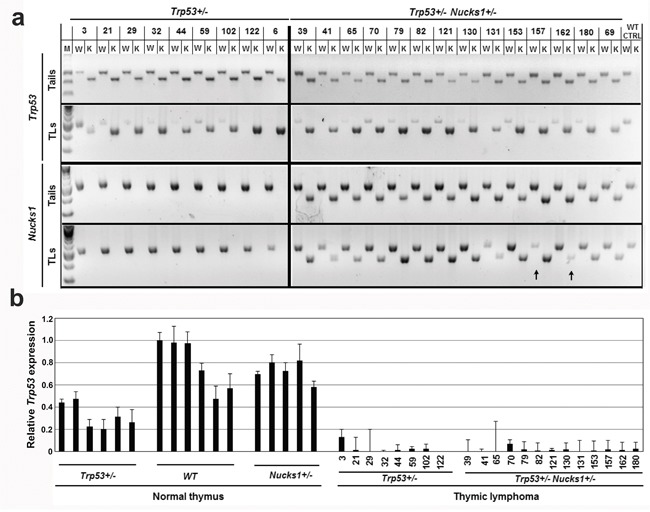
*Nucks1* is haplo-insufficient in suppressing IR-induced TL **a.** Agarose gels to show the results from semi-quantitative PCR analysis to detect LOH at *Trp53* and *Nucks1*. Genomic DNAs from tail and TL of the same mouse were used as template. Arrows indicate LOH of the *Nucks1* wild type and knockout alleles. M: molecular weight marker; W: wild type allele; K: knockout allele; WT CTRL: tail and thymus of a sham-irradiated 129S1/SvImJ mouse. **b.** Results from TaqMan qRT-PCR assays to assess *Trp53* expression in normal thymi of *Trp53*+/−, wild type (WT) and *Nucks1*+/− mice, and in IR-induced TLs of *Trp53*+/− and *Trp53+/−Nucks1+/−* mice.

TLs that arose from *Trp53*+/− and *Trp53*+/− *Nucks1*+/− mice showed LOH of the wild type *Trp53* allele in 8/9 and 14/14 cases, respectively (Figure 2a). Only one TL (TL-3) from a *Trp53*+/− mouse maintained the *Trp53* wild type allele, but showed greatly reduced expression of *Trp53* mRNA (Figure 2b). In contrast, LOH of the *Nucks1* wild type allele was rare and detected in one TL (TL-157; *Trp53*+/− *Nucks1*+/−) (Figure 2a). LOH of the mutant *Nucks1* allele in TLs of *Trp53*+/− *Nucks1*+/− mice was also rare, and occurred in 1/14 cases only (TL-162; Figure 2a).

Activation of NOTCH1 by intragenic deletion of the *Notch 1* exon 1 is common in lymphomas and irradiated thymi [26–28]. However, it was not clear whether deletion-based NOTCH1 activation also played a role in the radiation-induced TLs in *Trp53*-deficient mice, and if it was dependent on *Nucks1* status. We found that *Notch1* type 1 deletions were common in TLs from both cohorts, and that their frequencies were similar in TLs from *Trp53*+/− mice (8/9) and *Trp53*+/− *Nucks1*+/− mice (12/14; Supplementary Figure S3). These results suggest that, in the context of *Trp53* deficiency and independent of *Nucks1* status, deletion-based NOTCH1 activation is an important event during IR-induced T-cell lymphomagenesis.

### *Nucks1* expression is up-regulated in most TLs from *Trp53*+/− *Nucks1*+/− mice

**Figure 3:**
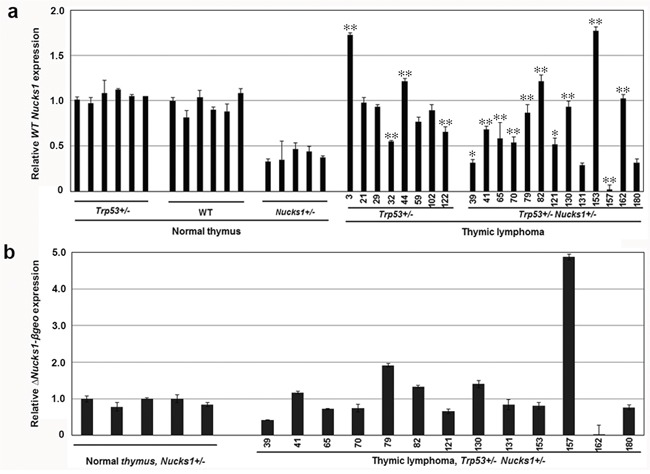
*Nucks1* mRNA expression in normal thymi and TLs **a.** Results from TaqMan qRT-PCR assays to assess *Nucks1* expression in normal thymi of *Trp53*+/−, wild type (WT) and *Nucks1*+/− mice, and in IR-induced TLs of *Trp53*+/− and *Trp53+/−Nucks1+/−* mice. *, *P*<0.05; **, *P*<0.01 (for *Nucks1* expression in TL of *Trp53*+/− mice compared to normal thymus of *Trp53*+/− mice, and for *Nucks1* expression in TL of *Trp53*+/− *Nucks1*+/− mice compared to normal thymus of *Nucks1*+/− mice). Note, that 9/13 TLs from *Trp53*+/− *Nucks1*+/− mice show significantly increased *Nucks1*, and 2/13 TLs from *Trp53*+/− *Nucks1*+/− mice show significantly decreased *Nucks1*. **b.** Results from TaqMan qRT-PCR assays to assess *ΔNucks1-βgeo* in normal thymi, and in IR-induced TLs of *Trp53*+/− *Nucks1*+/− mice.

We examined if *Nucks1* mRNA levels were altered in TLs from *Trp53*+/− *Nucks1*+/− mice. In normal thymi, wild type *Nucks1* mRNA expression correlated well with genotype in that ~40% *Nucks1* mRNA was expressed in *Nucks1*+/− thymi compared to *Nucks1+/+* thymi, and *Trp53* heterozygosity did not affect *Nucks1* mRNA expression (Figure 3a). *Nucks1* mRNA expression was higher than in normal *Nucks1+/+* thymi in 2/8 TLs (TL-3, TL-44) from *Trp53*+/− mice, but overall not significantly different between both groups (Figure 3a; *P*=0.703; Mann-Whitney test). The means of the dCTs for *Nucks1* to *Gapdh* were 2.79 and 2.78 for normal wild type thymi and for TLs of *Trp53*+/− mice, respectively. *Nucks1* expression in TLs from *Trp53*+/− mice was moderately decreased when compared to normal thymi from *Trp53*+/− mice (*P*=0.016; Mann-Whitney test). The means of the dCTs for *Nucks1* to *Gapdh* were 2.61 and 2.78 for normal thymi and TLs of *Trp53*+/− mice, respectively. However, *Nucks1* mRNA expression was significantly elevated in 9/13 TLs from *Trp53*+/− *Nucks1*+/− mice when compared to normal thymi of *Nucks1*+/− mice (*P*<0.0001; Mann-Whitney test; Figure 3a). The means of the dCTs for *Nucks1* to *Gapdh* were 4.04 and 3.29 for normal thymi from *Nucks1*+/− mice and for TLs from *Trp53*+/− *Nucks1*+/− mice, respectively. These results suggest that the up-regulation of *Nucks1* transcript is a frequent event in IR-induced TLs from *Trp53*+/− *Nucks1*+/− mice. *Nucks1* expression in TLs from *Trp53*+/− mice compared to TLs from *Trp53*+/− *Nucks1*+/− mice was significantly different (*P*=0.0072; Mann-Whitney test), with mean dCTs for *Nucks1* to *Gapdh* of 2.78 and 3.29, respectively.

We then examined the *ΔNucks1-βgeo* mRNA expression in normal thymi from *Nucks1*+/− mice and in TLs from *Trp53*+/− *Nucks1*+/− mice. In most TLs that arose from *Trp53*+/− *Nucks1*+/− mice, *ΔNucks1-βgeo* mRNA expression was not elevated when compared to normal thymi from *Nucks1*+/− mice (Figure 3b). TL-79 showed an ~2-fold increase in *ΔNucks1-βgeo mRNA* expression. TL-157 showed an ~5-fold increase in *ΔNucks1-βgeo* mRNA expression and TL-162 showed no *ΔNucks1-βgeo* mRNA expression, likely from gene conversion events involving gain and loss of the *Nucks1* knockout allele, respectively (see Figure 2a). In support of these findings, *Nucks1* mRNA expression was essentially zero in TL-157, but was ~2–fold increased in TL-162 when compared to normal *Nucks1*+/− thymi (Figure 3a).

**Figure 4:**
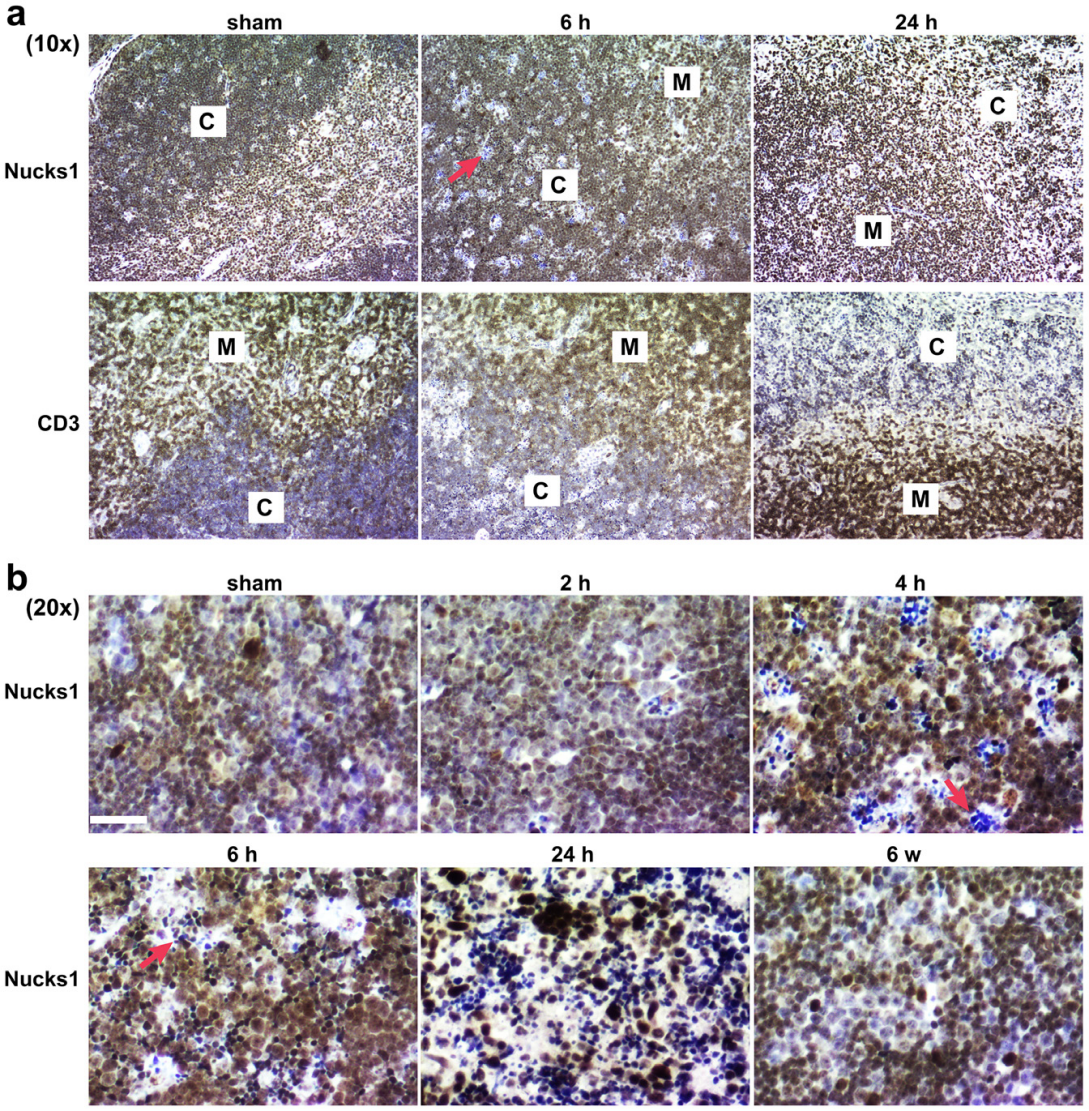
IR-induced cell death within the thymic cortex Five-week old wild type 129S1/SvImJ mice were either sham-treated or irradiated with 4 Gy TBI. CD3 and NUCKS1 expression was examined by IHC in FFPE sections of isolated thymi fixed at the times indicated. **a.** At 10× magnification, to show NUCKS1 and CD3 staining (brown) in both the cortex (C) and the medulla (M). **b.** At 20× magnification, to show NUCKS1 staining (brown) in the cortex. Red arrows: indicate apoptotic cells. Hematoxylin: nuclei. Scale bars: 200 μm.

To test if NUCKS1 protein levels change in wild type mice exposed to IR, five-week old wild type 129S1/SvImJ mice were either sham-irradiated or exposed to 4 Gy TBI, and thymi were harvested and fixed for immunohistochemistry (IHC) at 2 h, 4 h, 6 h, 24 h and 6 weeks post TBI. Cell death was observed within defined areas of the cortex as early as 4-6 h post treatment (Figure 4). Viable T-cells in the cortex overall expressed higher levels of NUCKS1 protein at 4-6 h post IR exposure than T-cells in sham-irradiated thymi, or in thymi that were recovered 6 weeks post IR exposure (Figure 4b). At 24 h post exposure, the vast majority of T-cells in the thymus cortex were dead, and viable T-cells with increased NUCKS1 were distributed mostly throughout the medulla (Figure 4a). These results suggest that NUCKS1 protein expression increases at early times post IR exposure in thymocytes of irradiated mice.

### Analyses of immunophenotypes and *Tcrb* clonality reveal differences in IR-induced TLs from *Trp53*+/− and *Trp53*+/− *Nucks1*+/− mice

**Figure 5:**
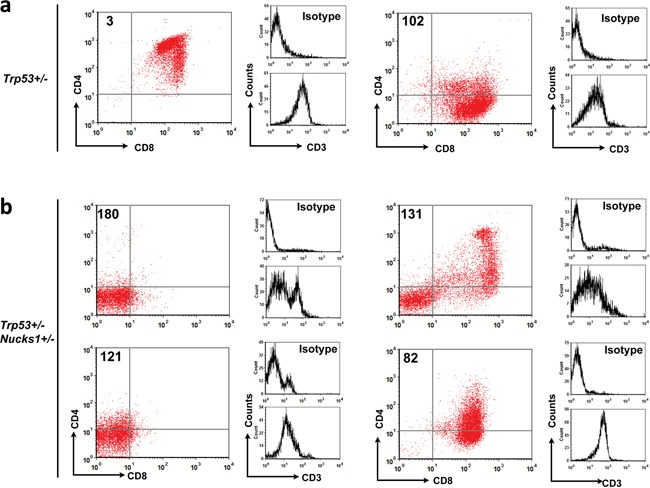
Immunophenotyping analyses of TL cells isolated from IR-induced TLs of *Trp53*+/− and *Trp53*+/− *Nucks1*+/− mice **a.** Primary TL cells from TLs of *Trp53*+/− mice were stained for CD4, CD8 and CD3 and analyzed by FACS. Representative 2-parameter dot plots showing that most primary TL cells from *Trp53*+/− mice were CD4+ CD8+ DP (TL-3) or CD8+ SP (TL-102) with moderate CD3 expression (see also Table 2). **b.** Same as in (a) for primary TL cells isolated from *Trp53*+/− *Nucks1*+/− mice. Note, that large compartments of CD4- CD8- DN TL cells were seen in TLs from *Trp53*+/− *Nucks1*+/− mice (TL-180, TL-121 and TL-131; see also Table 2).

We next analyzed the immunophenotypes of isolated TL cells from IR-induced TLs of *Trp53*+/− and *Trp53*+/− *Nucks1*+/− mice. CD3 expression for the majority of isolated TL cells from both cohorts was moderate, indicative of their immaturity (Figures 5a and 5b; Table 2). With the exception of TL-32, TL cells isolated from IR-induced TLs of *Trp53*+/− mice (8/9) were either CD4+ CD8+ double positive (DP) or CD8+ single positive (SP), or a mix of these two cell populations (Figure 5a; Table 2). TL-32 (from a *Trp53*+/− mouse) was the only rare case that showed a mixed population of CD4- CD8- double negative (DN) and uncommon CD4+ SP TL cells. In contrast, TLs of *Trp53*+/− *Nucks1*+/− double heterozygous mice frequently (5/8) contained CD4- CD8- DN TL cells (Figure 5b; Table 2). Among these, TL-180 and TL-121 were purely CD4- CD8- DN (Figure 5b), while TL-131, TL-82, TL-79 and TL-162 showed mixed populations of CD4- CD8- DN, CD4+ CD8+ DP and CD8+ SP TL cells (Figure 5b; Table 2).

**Table 2:** Summary: Immunophenotyping analyses of thymic lymphoma (TL) cells

Genotype	Mouse ID	TdT^1^	CD3^2^	CD4^3^	CD8^3^	*Tcra/d* transloc.	*Igh*transloc.
***Trp53*+/−**	3	+	+	+	+	N	Y
	29	+	+	− and mod	+	n.d.	n.d.
	32	+	mod	− and +	−	Y	Y
	44	+	mod/+	− and +	+	Y	Y
	44, P15^4^	n.d.	mod	−	+	n.d.	n.d.
	102	+	mod	−	+	Y	N
	102, P16^4^	n.d.	mod	−	+	n.d.	n.d.
	122	+	+ and −	+	+	Y	Y
	Ly-191^5^	+	+	−/mod	+	n.d.	n.d.
	Ly-200^5^	+	− and +	−	+	n.d.	n.d.
	Ly-233^5^	+	+	+ and −	+	n.d.	n.d.
***Trp53*+/− *Nucks1*+/−**	39	+	+	mod/+	+	N	N
	79	+	mod	−	− and mod	N	Y
	79, P15^4^	n.d.	−	−	−	n.d.	n.d.
	82	+	+	− and mod	+	N	N
	82, P15^4^	n.d.	+	−	+	n.d.	n.d.
	121	+	mod	−	−	n.d.	n.d.
	131	+	+/mod	− and +	− and +	N	N
	153	+	+/mod	+	+	Y	N
	162	+	+	−	− and mod	N	Y
	180	+	− and +	−	−	N	Y
***Trp53+/−*** ***Nucks1−/−***	573	n.d.	+	− and +	− and +	n.d.	n.d.
	573, P17^4^	n.d.	+	−	−	n.d.	n.d.
	576	n.d.	+	− and +	− and +	n.d.	n.d.

1 TdT was examined by IHC only.

2 CD3 immunophenotype was assessed by FACS using isolated primary TL cells, and by IHC using FFPE sections of TLs.

3 CD4 and CD8 were examined by FACS using isolated primary TL cells.

4 Established primary TL cell lines by sub-culturing: cells were split 1:10 ratio to maintain growth at a density ~5 × 10e5 cells/ml and counted as one passage.

5 These cell lines were derived from TLs of **Trp53+/−** 129S1/SvImJ mice irradiated with 4Gy TBI.

Definitions: “-”: negative; “mod”: positive, but with moderate intensity when compared to “+”; “+”: positive with good intensity; n.d.: not determined.

We also analyzed the immunophenotypes of TL cells isolated from two IR-induced TLs (TL-573 and TL-576) of *Trp53*+/− *Nucks1*−/− mice (further backcrossed to 129S1/SvImJ mice (*i.e*., F6 generation)). Both showed mixed populations of CD4- CD8- DN and CD4+ CD8+ DP TL cells (Table 2). Note, there was no difference in normal thymocyte maturation between sham-irradiated *Trp53*+/− and *Trp53*+/− *Nucks1*+/− mice (Supplementary Figure S4). Taken together, these results suggest that TLs in *Trp53*+/− *Nucks1*+/− mice originated from less mature thymocytes than TLs in *Trp53*+/− mice.

**Figure 6:**
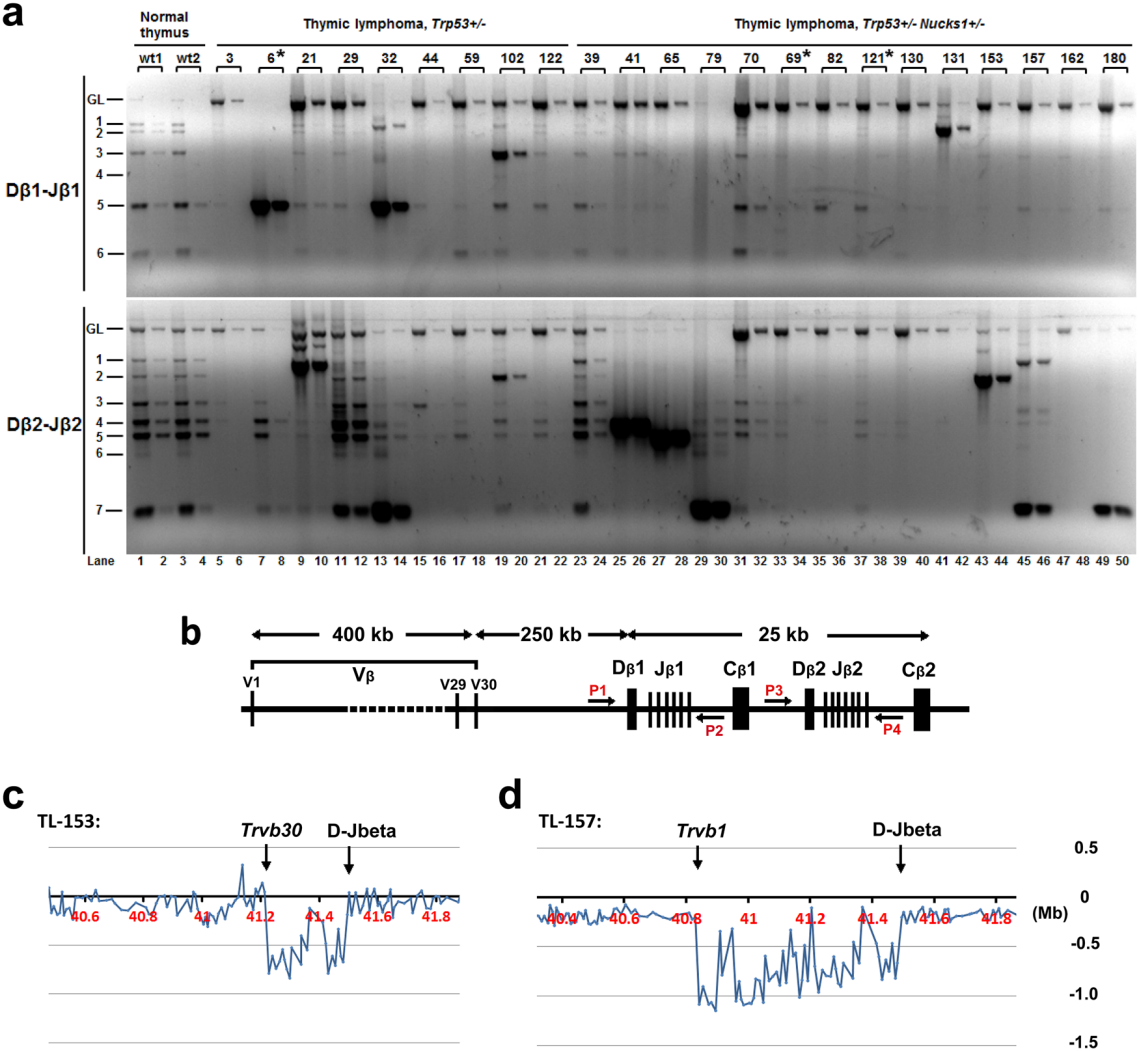
Analyses of Dβ-Jβ clonality at *Tcrb* in IR-induced TLs of *Trp53*+/− and *Trp53*+/− *Nucks1*+/− mice **a.** Representative agarose gels from semi-quantitative PCR analyses to assess Dβ1-Jβ1 and Dβ2-Jβ2 rearrangements at *Tcrb* using primer pairs P1 and P2 (Dβ1-Jβ1) and P3 and P4 (Dβ2-Jβ2), respectively, as show in (b). Fifty (odd lanes) and 5 ng (even lanes) of genomic DNA isolated from the thymi of normal wild type mice (wt1, wt2) and from IR-induced TLs of mice, with genotypes as indicated, were used. Asterisks: indicate TLs not analyzed by array-CGH. **b.** Schematic of the mouse *Tcrb* locus with relative genomic positions of the Vβ segments and Dβ-Jβ-Cβ clusters, and of PCR primers (P1, P2, P3 and P4). **c, d.** *Tcrb* rearrangements were also assessed by array-CGH analysis (representative expanded array-CGH profiles for TL-153 and TL-157 are shown). Note: For all TLs with genomic loss at *Tcrb*, the regions of loss spanned the *Vβs* and *Dβ-Jβ* clusters, indicative of *Vβ* to *Jβ1* or *Vβ* to *Jβ2* rearrangements. Minimal genomic loss was found in TL-153, as shown in **c.**, which included *Trvb30* segment, and maximal genomic loss was detected in TL-153, as shown in (d), which included the *Trvb1* segment.

PCR was used to test for *Dβ-Jβ* clonality at *Tcrb*. In *Trp53*+/− mice, 2/9 TLs (TL-6, TL-21) showed monoclonal expansion and 3/9 TLs (TL-29, TL-32 and TL-102) showed oligoclonal expansion (Figure 6a and 6b; Supplementary Table S1). As TL-29, TL-32 and TL-102 showed more than two predominant PCR products, likely these TLs originated from more than two cells. In contrast, 7/14 TLs from *Trp53*+/− *Nucks1*+/− mice showed clonal expansion (TL-41, TL-65, TL-79, TL-131, TL-153, TL-157 and TL-180), all of which showed just one or two predominant PCR products (Figure 6a).

**Figure 7:**
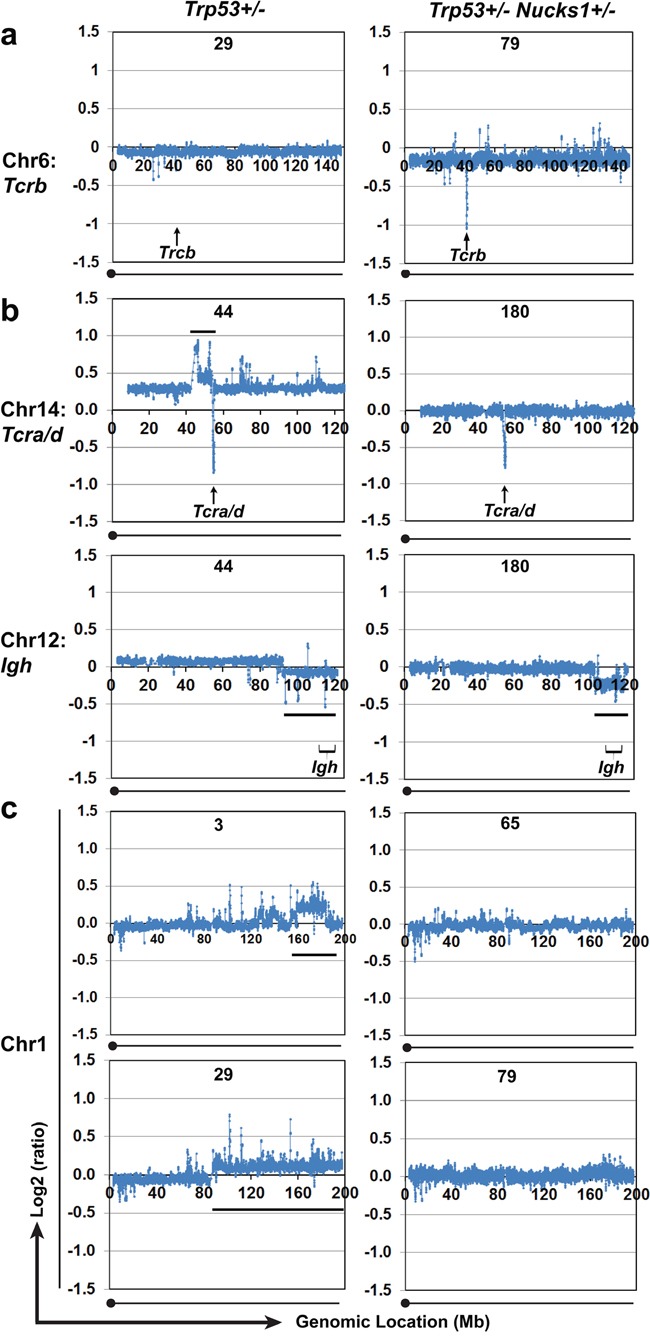
Array-CGH analyses show CNVs in chromosomes 6, 14, 12 and 1 in TLs from *Trp53*+/− and *Trp53*+/− *Nucks1*+/− mice **a.** Array-CGH profiles of chromosome 6 to show differences in genomic loss at *Tcrb* between TLs from *Trp53*+/− (T-29) and *Trp53*+/− *Nucks1*+/− (TL-79) mice. Extensive genomic loss at *Tcrb* was detected in the majority (10/12) of TLs from *Trp53*+/− *Nucks1*+/− mice, but only in 1/8 TLs from *Trp53*+/− mice (TL-21; Supplementary Figure S5). **b.** Array-CGH profiles to show CNVs in chromosomes 14 (upper panels) and 12 (lower panels) in TLs from one *Trp53*+/− (TL-44) and one *Trp53*+/− *Nucks1*+/− (TL-180) mouse. Black horizontal lines indicate the regions of gain centromeric to *Tcra/d* (chromosome 14) and the telomeric regions of loss including *Igh* (chromosome 12). **c.** Array-CGH profiles of chromosome 1 to show differences in CNVs between TLs from *Trp53*+/− (TL-3, TL-29) and *Trp53*+/− *Nucks1*+/− (TL-65, TL-79) mice. Gain of the telomeric region of chromosome 1 was detected in ~50% of TLs from *Trp53*+/−, but never in TLs from *Trp53*+/− *Nucks1*+/− mice.

Clonality at *Tcrb* was confirmed by testing for *Tcrb* locus copy-number variations (CNVs) using array-CGH. Consistent with our results for *Dβ-Jβ* clonality, monoclonal *Dβ-Jβ* rearrangements (as detected by PCR) showed extreme loss within *Tcrb* by array-CGH (*e.g*., TL-21 (*Trp53*+/−), and TL-41, TL-65, TL-79, TL-131, TL-153, TL-157, TL-180 (all *Trp53*+/− *Nucks1*+/−); Figure 6c and 6d, Figure 7a and Supplementary Figure S5). Mild loss of *Tcrb* was detected in TL-29 (*Trp53*+/−; Figure 7a), and in TL-32 and TL-102 (*Trp53*+/−; data not shown), reflecting their oligoclonal properties. TL-82 and TL-162 (*Trp53*+/− *Nucks1*+/−) showed extreme loss within *Tcrb* by array-CGH (Supplementary Figure S5), but clonal expansion of *Dβ-Jβ* events was not detected (see Figure 6a). The reasons for this are unclear at this point. However, it is possible, that complex rearrangements within *Tcrb* may have occurred, not detectable by the primers used for *Dβ1-Jβ1* and *Dβ2-Jβ2* recombination events here.

### *Trp53*+/− *Nucks1*+/− mice succumb to IR-induced TLs with clonal translocations involving *Igh* but not *Tcra/d*

We asked whether both cohorts of mice would harbor distinct clonal translocations, and if those would involve the *Tcra/d* and *Igh* loci [29, 30]. To do so, we analyzed array-CGH plots for translocation patterns involving *Tcra/d* and *Igh*. In *Trp53*+/− mice, typical patterns of centromeric amplification coupled with *Tcra/d* loss were seen in 3/8 TLs (TL-44 (Figure 7b), and in TL-32 and TL-102, and an atypical pattern with gain telomeric to *Tcra/d* was observed in TL-122 (Supplementary Figure S6A)). These results show that around half of the TLs from *Trp53*+/− mice harbored clonal translocations involving *Tcra/d*. In contrast, TLs from *Trp53*+/− *Nucks1*+/− mice, which contained mixed CD4- CD8- DN TL cell populations (TL-79, TL-131 and TL-162) or were solely CD4- CD8- DN (TL-121 and TL-180), clonal translocations involving the *Tcra/d* were rare and detected in TL-65 and TL-153 only (Supplementary Figure S6A). Interestingly, TL cells isolated from TL-153 were CD4+ CD8+ DP without any CD4- CD8- DN TL cells (Table 2). TL-65, unfortunately, was unavailable for surface staining due to poor cell viability after isolation.

We next examined *Igh*-involving translocations in TLs from both Trp53*+/−* and *Trp53*+/− *Nucks1*+/− mice. In *Trp53*+/− mice, loss of the region telomeric to *Igh* was detected in TL-44 (Figure 7b), and in TL-3, TL-32 and TL-122 (Supplementary Figure S6B). In *Trp53*+/− *Nucks1*+/− mice, loss of the region telomeric to *Igh* was detected in TL-180 (Figure 7b) and in TL-65, TL-79, TL-157 and TL-162 (Supplementary Figure S6B), suggesting that translocations involving the *Igh* locus occurred in both cohorts with similar frequencies.

### Array-based CGH analysis shows that CNVs in TLs from *Trp53*+/− and *Trp53*+/− *Nucks1*+/− mice are both specific and overlapping

Array-based CGH analysis was used to examine genome-wide CNVs in TLs from *Trp53*+/− and *Trp53*+/− *Nucks1*+/− mice. The overall patterns of genome-wide CNVs were similar between TLs from both cohorts, including changes at *Igh and Tcrg* (Figure 7b, Supplementary Figure S6B; data not shown). In addition, chromosome regions that contain the *Ikaros* [31, 32] and *Pten* genes [33, 34] were lost frequently in TLs from both *Trp53*+/− and *Trp53*+/− *Nucks1*+/− mice (data not shown). CNVs, other than at *Tcra/d* and *Tcrb*, that were more specific to one but not the other cohort were also detected. For example, gains in the telomeric arm of chromosome 1, reported to correlate tightly with cancer occurrence [35], were observed in ~50% of the TLs from *Trp53*+/− mice (TL-3, TL-29 (Figure 7c), TL-32 and TL-102 (Supplementary Figure S6C)). However, none of the TLs from *Trp53*+/− *Nucks1*+/− mice showed gain in the telomeric arm of chromosome 1 (Figure 7c; data not shown).

### TLs from *Trp53*+/− *Nucks1*+/− mice show increased frequency of infiltration

**Figure 8:**
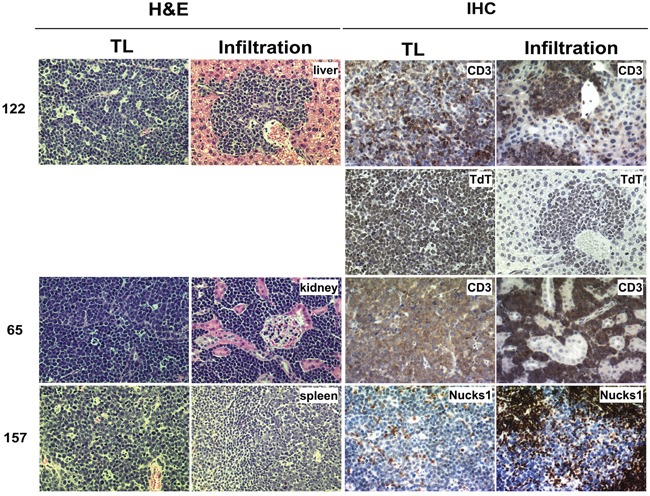
TLs from *Trp53*+/− *Nucks1*+/− mice infiltrate distant organs more frequently than TLs from *Trp53*+/− mice TL cells infiltrating target organs were surrounded by blood vessels and were discriminated from the target tissue based on H&E staining (left). Further evidence of infiltration was obtained by IHC using anti-CD3 (TL-122 and TL-65), anti-TdT (TL-122) and anti-NUCKS1 (TL-157) antibodies. TL-122: *Trp53*+/−; TL-65 and TL-157: *Trp53*+/− *Nucks1*+/−.

In 1/10 of the *Trp53*+/− mice, TL cells (*i.e.*, TL-122) were found to infiltrate the liver (Figure 8; Supplementary Table S2). In contrast, in 4/14 *Trp53*+/− *Nucks1*+/− mice TL cells were found to infiltrate distant organs, including kidney (TL-65 and TL-162), spleen (TL-157 and TL-131), and liver (TL-162) (Figure 8; Supplementary Table S2). To confirm these observations, additional *Trp53*+/− and *Trp53*+/− *Nucks1*+/− mice (further backcrossed to 129S1/SvImJ mice (F6 generation, with an average genetic background of > 99% 129S1/SvImJ)) were exposed to 4 Gy TBI. At the time the mice became moribund from TLs, mice were euthanized, assessed for the occurrence of TL and for infiltrating TL cells to distant organs. Similar to the observations for the F2 generation, increased infiltration of TL cells to distant organs was detected in *Trp53*+/− *Nucks1*+/− mice (4/13; liver, kidney and spleen) compared to *Trp53*+/− mice (1/10; liver only; Supplementary Table S2).

## DISCUSSION

Environmental factors, including exposure to IR, are recognized as exogenous risk factors that induce genetic changes to drive carcinogenesis. Mice harbor many single-nucleotide polymorphisms and CNVs similar to those observed in humans [36, 37], and mouse models are powerful tools for the identification of alleles associated with susceptibility or resistance to carcinogenesis.

The effects of *Nucks1* inactivation on carcinogenesis in mice had not been investigated. In human cells, however, NUCKS1 deficiency after siRNA-mediated knockdown impairs DNA damage signaling, DSB repair and genome stability [14], suggesting that this protein may function as a tumor suppressor. Here, we investigated the phenotypic consequences of partial *Nucks1* deficiency and the susceptibility of these mice to radiation carcinogenesis. As the constitutive inactivation of HR genes in mice frequently leads to embryonic lethality [24, 25, 38–40], we chose to conduct our studies in a *Nucks1+/− Trp53+/−* double heterozygous context. Using a *Trp53*+/− mouse model that is prone to radiation-induced TL [32, 34, 41, 42], we found that heterozygous *Nucks1* synergistically accelerated radiation lymphomagenesis and also led to other changes in the associated tumor spectrum. Importantly, IR-induced TLs that arose in double heterozygous *Nucks1+/− Trp53+/−* mice developed at earlier lineage stage and more frequently led to the infiltration of distant organs than TLs in single heterozygous *Trp53*+/− mice. We speculate that, in our mouse model, *Trp53* loss itself (as observed in both cohorts to essentially full extent) does not suffice to fully drive invasive migration, in accord with what has been reported by others [43, 44]. Our findings for *Trp53*+/− mice also are consistent with our unpublished results (J.-H. Mao) that show that IR-induced TLs from *Trp53*+/− mice rarely infiltrate distant organs (*e.g*., liver, kidney and spleen). In contrast, TLs from *Nucks1+/− Trp53+/−* mice show a much greater propensity to infiltrate distant organs, and it will be important in the future to investigate the additional molecular determinants involved in this difference.

Many tumors contain chromosomal translocations and deletions (resulting from mis-repaired or unrepaired DSBs) leading to the activation of oncogenes and/or inactivation of tumor suppressor genes [45]. As such, DSBs likely play a major role in driving malignant transformation. Notably, NUCKS1 deficiency in human cells has been linked to a DSB repair defect [14], and inactivation of NHEJ and HR factors in mice leads to increased tumor burden, frequently resulting from immature T-cell lymphomas [30, 46, 47]. Defective repair of IR-induced DSBs can lead to TLs associated with translocations, deletions and CNVs [30, 48–51], similar to the findings reported here. Specifically, we show that TLs in *Trp53*+/− mice predominantly exhibited CNVs indicative of chromosomal translocations at *Tcra/d*, as reported previously [52], whereas TLs in *Trp53*+/− *Nucks1*+/− mice predominantly exhibited CNVs indicative of chromosomal translocations at *Igh*. Combined with the observation that the majority of TLs from *Trp53*+/− mice were CD4+ CD8+ DP or CD8+ SP, we suggest that IR-induced TLs in *Trp53*+/− mice largely originated at the DP lineage stage, the stage during which *Tcra/d* recombination occurs. In contrast, in *Trp53*+/− *Nucks1*+/− mice, 5 of the 8 analyzed TLs contained mixed CD4- CD8- DN TL cell populations or were purely CD4- CD8- DN, suggesting that a large proportion of TLs in *Trp53*+/− *Nucks1*+/− mice originated earlier than the DP lineage stage. Among the TLs that contained CD4- CD8- DN TL cell populations or that were purely CD4- CD8- DN, clonal translocations involving *Tcra/d* were not detected. Interestingly, the presence of CD4- CD8- DN cells detected in this study in TLs from *Trp53*+/− *Nucks1*+/− mice is similar to that reported in TLs derived from mice with somatic mutation of *Trp53* in hematopoietic stem cells [53], and could potentially be related to the disruption of the T cell checkpoint pathways through PD-1 expression [54].

Our results suggest that ~ 50% of TLs in *Trp53*+/− *Nucks1*+/− mice originated from one or two cells, while TLs in *Trp53*+/− mice largely appear to have originated from several pre-cancerous cells. Moreover, TLs in *Trp53*+/− *Nucks1*+/− mice frequently developed from less mature thymocytes than those in *Trp53*+/− mice. The heterogeneous expression of CD4 and CD8 in TL-79 and TL-131 from two double heterozygous mice suggests that these TLs originated from single thymocytes and accumulated subsequent changes during tumor progression.

It has been well established that IR almost exclusively induces LOH of the *Trp53* wild type allele in TLs derived from *Trp53*+/− mice [41, 55], in line with the results presented here. However, the exact course of events taken during TL development for loss of the wild type *Trp53* allele to occur is unclear. Notably, LOH can be caused by several mechanisms, including mitotic recombination, mitotic non-disjunction and also by multi-locus deletion events. We speculate that partial NUCKS1 deficiency may lead to the accelerated loss of the *Trp53* wild type allele in TLs from *Trp53*+/− *Nucks1*+/− mice, that compared to TLs from *Trp53*+/− mice arise at an earlier stage during T-cell development (*i.e*. show increased DN T-cell populations), predominate in *Tcrb* mono- and bi-clonality, and show relatively higher fractions of *Igh*- than of *Tcra/d*-associated translocation events, suggestive of their overall dependency on fewer genetic changes for tumor formation. Interestingly, *Trp53*+/− *Nucks1*+/− mice also developed primary lung epithelial tumors that were never observed in *Trp53*+/− single heterozygous mice, in accord with a previous report investigating *Trp53*+/− mice only [52]. In contrast, loss of the remaining *Nucks1* wild type allele was rare in *Trp53*+/− *Nucks1*+/− mice, suggesting linkage of murine *Nucks1* to essential loci on chromosome 1.

**Figure 9:**
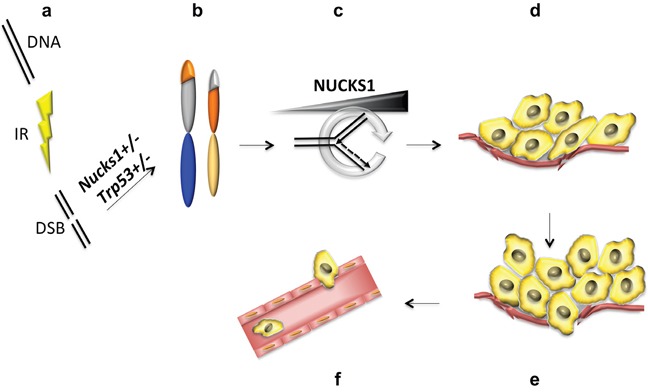
Model to explain the role of NUCKS1 in tumor initiation and progression **a, b.** Partial deficiency of NUCKS1 leads to a defect in DNA double-strand break (DSB) repair and to genome rearrangements (*i.e*., translocations) after ionizing radiation (IR) exposure. **c.** Tumor initiating cells experience increased replication stress, as described previously [60–62], which potentially may be ameliorated by up-regulation of NUCKS1 expression. **d.** High levels of NUCKS1 provide a selective advantage and promote TL growth, and **e.** invasion of the tumor border leading to **f.** intravasation of TL cells into the circulatory system.

*NUCKS1* expression is reduced in adult T-cell leukemia-lymphoma and in childhood acute lymphoblastic leukemia [22, 23]. However, the human *NUCKS1* gene also belongs to a group of co-expressed genes located on chromosomal region 1q32.1 that is amplified in some breast cancers [16, 17, 56, 57], and in other cancers [18, 19, 21, 23, 35, 58, 59]. Interestingly, in mouse lung adenocarcinoma, amplification of a similar region on mouse chromosome 1, which results in elevated expression of several genes including *Nucks1*, correlates with tumor invasiveness [58]. It is interesting to note that we find increased expression (relative to normal thymi from *Nucks1*+/− mice) of the wild type *Nucks1* transcript in 9/13 of the TLs isolated from *Trp53*+/− *Nucks1*+/− mice. We speculate that, within a subset of IR-induced TLs in *Trp53*+/− *Nucks1*+/− mice, increased expression of *Nucks1*/NUCKS1 may be associated with a selective advantage during tumor initiation and development, enabling early neoplastic cells to overcome replication stress during lymphomagenesis (Figure 9), thereby limiting DSBs resulting from collapsed replication forks [60, 61]. Of note, NUCKS1 plays an important role in mitigating replication stress in human cells, as we [14] and others [21] have shown. It is also possible that, in TLs from *Trp53*+/− *Nucks1*+/− mice, *Nucks1* expression is upregulated as part of the activated DDR network during tumorigenesis, as described previously for clinical specimens from different stages of human tumors [62].

We have shown that there is a direct link between *Nucks1* status and radiation carcinogenesis in mice. The results of our investigation are relevant to many published reports [16–19, 21–23, 35, 56–59] that indirectly have described an association between *NUCKS1*/NUCKS1 and several human cancer types. In a different genetic background, *Nucks1*−/− mice with wild type *Trp53* were reported to exhibit decreased insulin signaling and increased body weight/fat mass along with impaired glucose tolerance and reduced insulin sensitivity [63], related to the role of NUCKS1 in the hypothalamus [64]. These studies demonstrate that NUCKS1 can function as a tissue-specific transcriptional regulator of the insulin receptor, critical for insulin signaling and consequent peripheral metabolic activities. Interestingly, the DDR intersects the insulin-IGF1-PI3K-AKT pathway at many points [5]. While Qiu *et al*. [63, 64] did not expose their *Nucks1*−/− mice to DNA damaging agents and did not investigate the direct phenotypic consequences of persistent or mis-repaired DNA damage in these mice, persistent and repetitive DNA damage has been proposed to alter insulin-IGF1 signaling, thereby contributing to diabetes and other age-associated metabolic disorders [5]. In future investigations it therefore will be important to further dissect the role of the NUCKS1 protein in metabolic syndrome and in cancer avoidance to improve both diagnosis and targeted therapy of these prevalent ailments in humans.

## MATERIALS AND METHODS

### Irradiations

Five week-old F2 *Trp53*+/− and *Trp53*+/− *Nucks1*+/− littermates (Supplementary Materials and Methods) were exposed to 4 Gy TBI using a 360 kVp X-ray machine (Precision X-ray Inc., North Branford, CT, USA). Necropsies were performed when mice appeared morbid, or at the end of the study (45 weeks post TBI). The Kaplan-Meier method was used to compare the tumor development between different genotypes. The study was carried out in strict accordance with the Guide for the Care and Use of Laboratory Animals of the National Institutes of Health.

### Array based-comparative genomic hybridization (array-CGH)

Array-CGH experiments were performed on two-color Agilent SurePrint G3 Mouse CGH Microarrays (4×180k; Ambry Genetics, Aliso Viejo, CA, USA). Microsoft Excel-based programs (XY Scatter) were used for ratio data normalization and profile presentation.

### Immunohistochemistry

Formalin-fixed paraffin embedded (FFPE) tumor tissues were used to generate 0.5 μm tissue sections (Mouse Pathology Core, UCSF, San Francisco, CA, USA). Sections were heated to 60°C for 30 min and deparaffinized using xylene. Heat-induced epitope retrieval was carried out using Antigen Retrieval Solution (Dako, Carpinteria, CA, USA). Sections were blocked with egg white solution (2/200 ml H_2_O) and 5% non-fat dried milk powder in PBST (PBS with 0.1% Tween-20) for 20 min at room temperature. Horseradish peroxidase (HRP) activity was quenched by incubating in 0.3% H_2_O_2_ diluted in 0.1% NaN_3_/PBS for 20 min at room temperature. Antibody incubations were carried out as described (VECTASTAIN; Vector Laboratories, Burlingame, CA, USA) using ImmPACT DAB peroxidase substrate (Vector Laboratories). Sections were then counterstained with hematoxylin, dehydrated and mounted with Permount mounting medium (Fisher Scientific, Waltham, MA, USA). Primary antibodies used were: anti-CD3e (Abcam, Cambridge, MA, USA; ab5690), anti-TdT (Abcam; ab85148), anti-NUCKS1 (Bethyl Laboratories, Montgomery, TX, USA; IHC-00303) and anti-β-galactosidase (Life Technologies, Carlsbad, CA, USA; AB_10055437). Secondary antibodies were biotinylated horse anti-mouse or anti-rabbit IgG (Vector Laboratories).

### Immunophenotyping of primary TL cells by flow cytometry

Primary TL cells were isolated from dissected murine TLs and cultured in RPMI-1640 medium with 10% FBS and 1% antibiotics/antimycotics. Freshly isolated or sequentially expanded TL cells were used for cell surface marker staining. The following fluorochrome-conjugated primary antibodies (BD Biosciences, San Jose, CA, USA) were used: PE-Cy7-CD3e, PE-CD4, and APC-CD8a. A FACSCalibur flow cytometer (BD Biosciences) with CellQuest Software (BD Biosciences) was used for data acquisition. Live cells were gated based on forward and side scatter. Data analyses were done using *FCS Express* 4 software package (De Novo Software, Los Angeles, CA, USA).

### PCR detection of *Tcrb* DJ rearrangements

*Tcrb* gene DJ rearrangements were analyzed as described [65]. Five and 50 ng of genomic DNA were used for semi-quantitative PCR to detect *Tcrb* D-J recombination. Primer sequences were as listed in Supplementary Table S1.

### Statistical analyses

Comparisons between different groups of mice for tumor-free survival were made using a log-rank test (SPSS Statistics, IBM Software). Comparisons of *Nucks1* expression between groups of mice were made using the Kruskal-Wallis test (SPSS Statistics, IBM Software) and Mann-Whitney test (Prism GraphPad). Otherwise, Fisher's exact or Student's t tests were used, as stated, to determine significance (Prism GraphPad).

For further Materials and Methods see **Supplementary Information**.

## SUPPLEMENTARY MATERIALS FIGURES AND TABLES


