# Supplementary material for: Nucks1 synergizes with Trp53 to promote radiation lymphomagenesis in mice
Source: Oncotarget. 2016 Aug 16;7(38):61874–89. doi: 10.18632/oncotarget.11297 (PMC5308697; doi:10.18632/oncotarget.11297)
Supplement: Supplementary file 1 [file oncotarget-07-61874-s001.pdf]

Nucks1 synergizes with *Trp53* to promote radiation lymphomagenesis in mice

SUPPLEMENTARY MATERIALS AND METHODS

Genotyping

For genotyping, genomic DNA was extracted from 0.3 cm tail biopsies or tumor tissues using standard procedures. Primers used for genotyping PCR are listed in Supplementary Table S1. For the *Trp53* locus, PCR was performed using primers *Trp53* F1 and *Trp53* R1 for the wild type allele, and primers *Trp53* F2 and *Trp53* R1 for the knockout allele. For the *Nucks1* locus, PCR was performed using primers a and b for wild type allele, and primers a and c for knockout allele.

Cell culture and growth

Primary mouse embryonic fibroblasts (MEFs) were isolated from E13.5 embryos using standard procedures. Briefly, hematopoietic tissues and the head were removed and the remaining embryos were minced into small pieces with scissors. Minced embryos were then digested with trypsin-EDTA (0.05%) at 37°C for 10 min and homogenized by pipetting up and down. Released single cell suspensions were cultured in DMEM medium (Invitrogen) supplemented with 10% heat-inactivated FBS (Sigma), 2 mM L-glutamine (Invitrogen), 1× NEAA (Invitrogen), 50 µM β-mercaptoethanol (Sigma), and 1% antibiotics/antimycotics (Invitrogen) in a low oxygen (3%) incubator at 10% CO_2 and 37°C. After reaching confluency, MEFs were trypsinized and harvested, and labeled as passage 0 for cryopreservation or passaged further.

Primary thymocytes or TL cells were isolated from normal thymus or TLs, respectively. Thymocytes were isolated by mincing a dissected normal thymus through the 45 µm cell strainer using the plunger of a syringe. For TL cells, dissected tumors were first cut into small pieces followed by digestion using collagenase/dispase solution (Roche) at 37°C for 30 min. Released tumor cells were serially passed through medical gauze sponges (Fisher Scientific), and then a 70 µm and a 45 µm cell strainer (BD Pharmingen). Cells were washed in 2% FBS in PBS several times and cultured in RPMI-1640 medium (Invitrogen) supplemented with 10% FBS, 50 µM β-mercaptoethanol (Sigma), 1% antibiotics/antimycotics (Invitrogen) and 10-20 U/ml hIL-2 (Sigma). Cell densities were maintained at 0.5-1×10^6 cells/ml.

TaqMan qRT-PCR

Total RNA was extracted from murine thymic lymphomas or normal thymi using TRIzol reagent (Thermo Fisher Scientific) and was purified using RNeasy mini spin columns (QIAGEN). RNA quality was analyzed using Agilent RNA 6000 Nano Kit (Agilent Technologies) on a 2100 BioAnalyzer (Agilent Technologies). RNA with a RNA index number (RIN) of 8.0 or higher was used for reverse-transcription. For each sample, 1-2 µg of total RNA was reverse-transcribed to cDNA using the High Capacity cDNA Reverse Transcription Kit (Applied Biosystems). Real time PCR reactions were performed using TaqMan assays (Applied Biosystems) and a 7500 Fast Real-Time PCR System (Applied Biosystems) for 40 cycles. The pre-labeled TaqMan Gene Expression Assays (Life Technologies) used were: *MmNucks1* (Mm01288571_m1; spans exons 5 and 6 which are not present in the *Nucks1* knockout allele), *LacZ* (Mr03987581_mr), *Trp53* (Mm01731290_g1) and *GAPDH* (Mm99999915_g1). Values were normalized to *GAPDH* ($2^{-\Delta CT}$).

X-gal staining

Primary MEFs grown to 50% confluence were washed with PBS twice and fixed in 2% paraformaldehyde/0.2% glutaraldehyde/0.01% NP-40 in PBS at room temperature for 15 min. Fixative was then removed and cells was rinsed twice in PBS rinse buffer containing 2 mM $MgCl_2$, 0.01% sodium deoxycholate and 0.02% NP-40. X-Gal staining buffer was prepared in PBS rinse buffer with 5 mM postassium ferricyanide, 5 mM postassium ferrocyanide and 1 mg/ml 5-bromo-4-chloro-3-indolyl-β-D-galactoside (X-Gal, Thermo Fisher Scientific). After incubation for 12-24 hours at 37°C in X-Gal staining buffer, monolayers were rinsed with PBS, and imaged using a using a Zeiss Axioskop with a 100× objective.

Notch1 Type 1 deletion assay

Notch1 Type 1 deletions were analyzed by PCR as previously described with minor modifications [1]. Briefly, genomic DNA was extracted from mouse thymic lymphoma tissue and primers Type1d_F (forward) and Type1d_R (reverse) spanning the *Notch1* Type 1 deletion, were used to detect the Type 1 deletion events by PCR.

Either 50 ng or 100 ng template DNA were used in the PCR reaction, containing 2 µl 10× PCR buffer, 0.16 µl 25 mM dNTPs each, 1 µl 5 µM primer each, 0.1 µL QIAGEN HS Taq (QIAGEN) in a 20 µl reaction. PCR cycles were: 15 min at 95°C, followed by 40 cycles of 30 s at 94°C, 45 s at 55°C and 1 min at 72°C. PCR products were analyzed on 2% agarose gels. To confirm that the PCR products resulted from *Notch1* Type 1 deletions, PCR products were purified and sequenced using forward and reverse PCR primers. Obtained sequences were annotated to the mouse genome database (GRCm38.p2, C57BL/6J).

SUPPLEMENTARY RESULTS

Characterization of the gene-trapped mouse *Nucks1* locus

To monitor the trapped *Nucks1* allele (-; K) in $Nucks1^{Gt(XG374)Byg}$ mice, PCR analysis was performed using DNA from tail tissue with primers (a and c) spanning the 5′ intron 4-vector junction (Supplementary Figures 1A-1B; Supplementary Table S1). *Nucks1*+/− F1 mice were crossed to obtain *Nucks1*−/− *mice* that we found to be viable. The expression of the wild type NUCKS1 and the mutant ΔNUCKS1-βgeo proteins was examined using protein extracts from mouse embryonic fibroblasts (MEFs) with different genotypes. The results showed good correlation of protein expression level to genotype (Supplementary Figure 1C). The β-galactosidase activity of ΔNUCKS1-βgeo was also assessed using X-Gal staining in primary MEFs. The intensities of X-Gal staining showed good correlation with *Nucks1* genotype (Supplementary Figure 1D).

SUPPLEMENTARY REFERENCE

1. Ashworth TD, Pear WS, Chiang MY, Blacklow SC, Mastio J, Xu L, Kelliher M, Kastner P, Chan S, Aster JC. Deletion-based mechanisms of Notch1 activation in T-ALL: key roles for RAG recombinase and a conserved internal translational start site in Notch1. Blood. 2010; 116:5455-5464.

SUPPLEMENTARY FIGURES AND TABLES

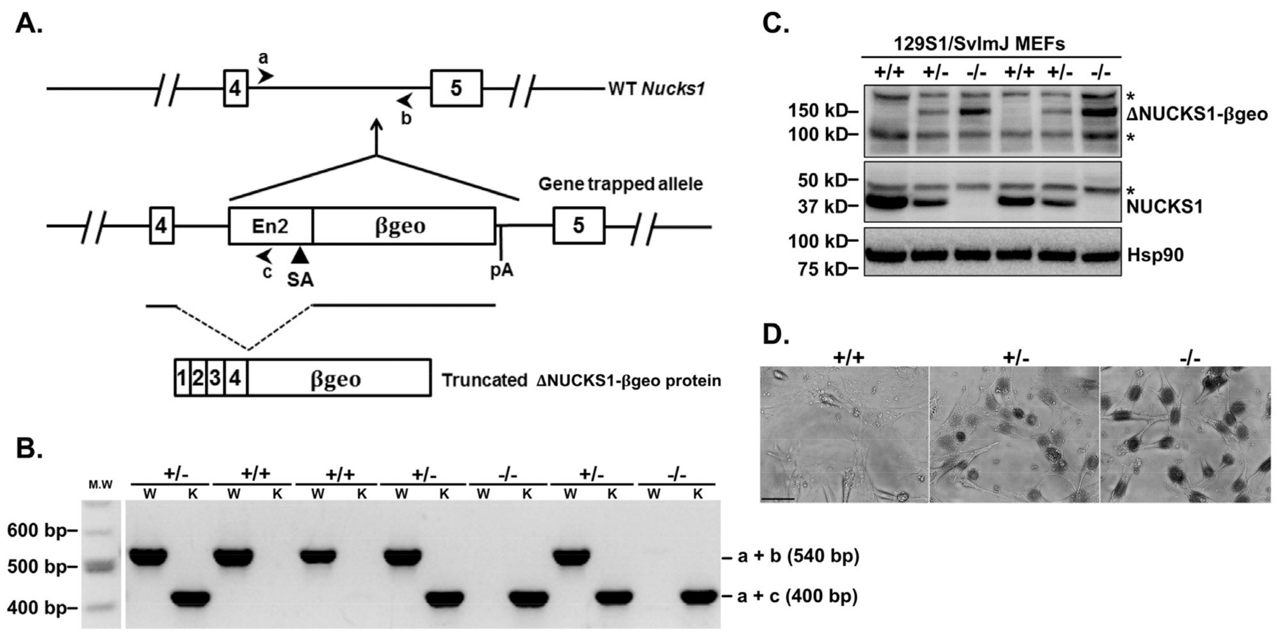

Supplementary Figure S1: Schematic and characterization of the trapped *Nucks1* allele in heterozygous and homozygous *Nucks1*$^{Gt(XG374)Byg}$ mice. A. Diagram of the gene-trapped *Nucks1* allele. pGT1Lxf was inserted into intron 4 of the *Nucks1* gene and contains the mouse *En2 intron 1* sequence, an *En2* exon/splice acceptor (SA) sequence, the ORF for *βgeo* (a fusion gene composed of *β-galactosidase* and *neomycin* genes) as selective maker, and a polyadenylation (pA) signal sequence. Expression of the gene-trapped allele results in a fusion protein composed of the first 4 exons of the *Nucks1* gene and of the *βgeo* gene. **B.** Results from PCR genotyping analysis using genomic DNA derived from tails and primers a and b to detect the wild type (+; W), and primers a and c to detect the mutant (-; K) *Nucks1* alleles. **C.** Western blot analysis of protein extracts obtained from mouse embryonic fibroblasts (MEFs) with genotypes as indicated. HSP90: loading control; asterisks: non-specific bands. **D.** X-Gal staining in MEFs with *Nucks1* genotypes as indicated. Scale bar: 50 µm.

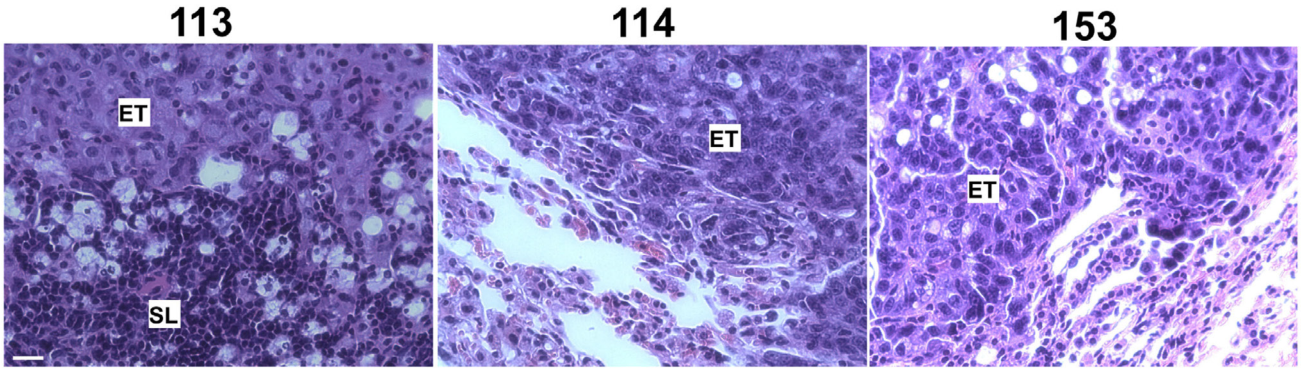

Supplementary Figure S2: Epithelial lung tumors in *Trp53*+/− *Nucks1*+/− mice. Unlike any of the *Trp53*+/− mice, a few *Trp53*+/− *Nucks1*+/− mice (113, 114, and 153) developed epithelial lung tumors in response to 4 Gy TBI. Representative micrographs from H&E stained lungs with primary epithelial tumors (ET) are shown. SL: splenic lymphoma (infiltrative). Scale bar: 20 µm.

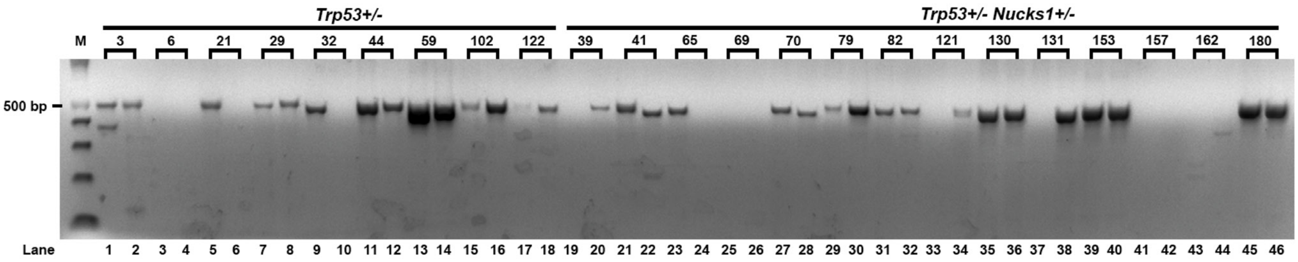

Supplementary Figure S3: Analysis of *Notch1* Type 1 deletion mutations in TLs from *Trp53*+/− and *Trp53*+/− *Nucks1*+/− mice. Representative agarose gel to show PCR products obtained to detect *Notch1* Type 1 deletion mutations in TLs from both *Trp53*+/− and *Trp53*+/− *Nucks1*+/− mice. One hundred ng (even lanes) and 50 ng (odd lanes) of genomic template DNA were used. In most TLs from both *Trp53*+/− (lanes 1-18) and *Trp53*+/− *Nucks1*+/− (lanes 19-46) mice a PCR product of ~500 bp, as a result of *Notch1* Type 1 mutation, was detected. Overall, no difference in the frequencies of *Notch1* Type 1 deletion mutations was observed comparing TLs from *Trp53*+/− mice and *Trp53*+/− *Nucks1*+/− mice.

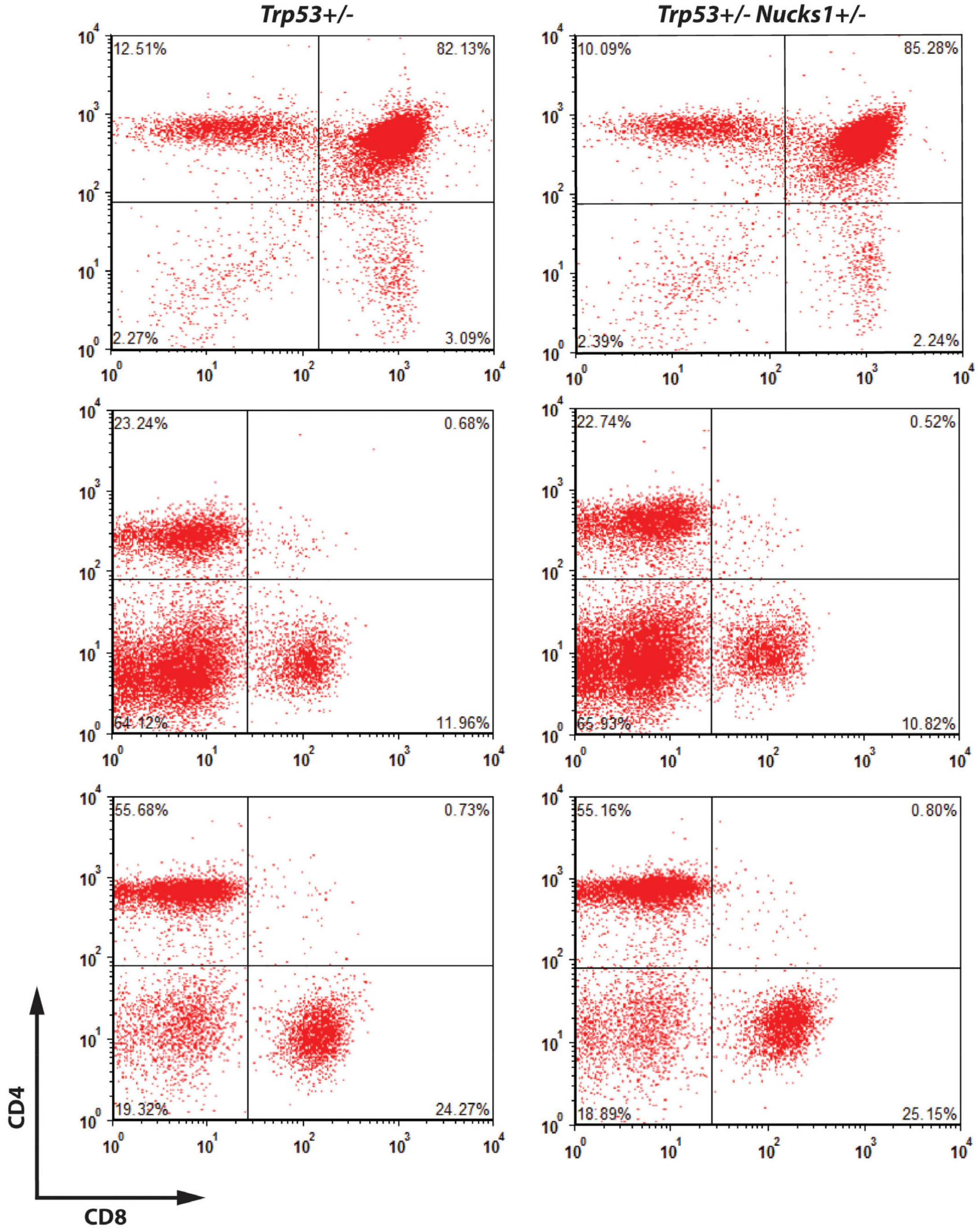

Supplementary Figure S4: No difference in T-cell maturation between *Trp53*+/− and *Trp53*+/−*Nucks1*+/− mice. Thymocytes, splenocytes and lymphocytes from peripheral lymph nodes were obtained from 6-week old mice. Shown are representative 2-parameter dot blots obtained after anti-CD4 and anti-CD8 staining and FACS analysis (see Materials and Methods), using isolated lymphocytes from thymus (upper panels), spleen (middle panels) and lymph nodes (lower panels). Note: Double-negative cell populations (lower left quadrant) in spleen (middle panels) and lymph nodes (lower panels) are B-cells. Data were collected from 4 *Trp53*+/− and 4 *Trp53*+/− *Nucks1*+/− mice from 2 different litters. No difference in T-cell maturation was detected between *Trp53*+/− and *Trp53*+/− *Nucks1*+/− mice.

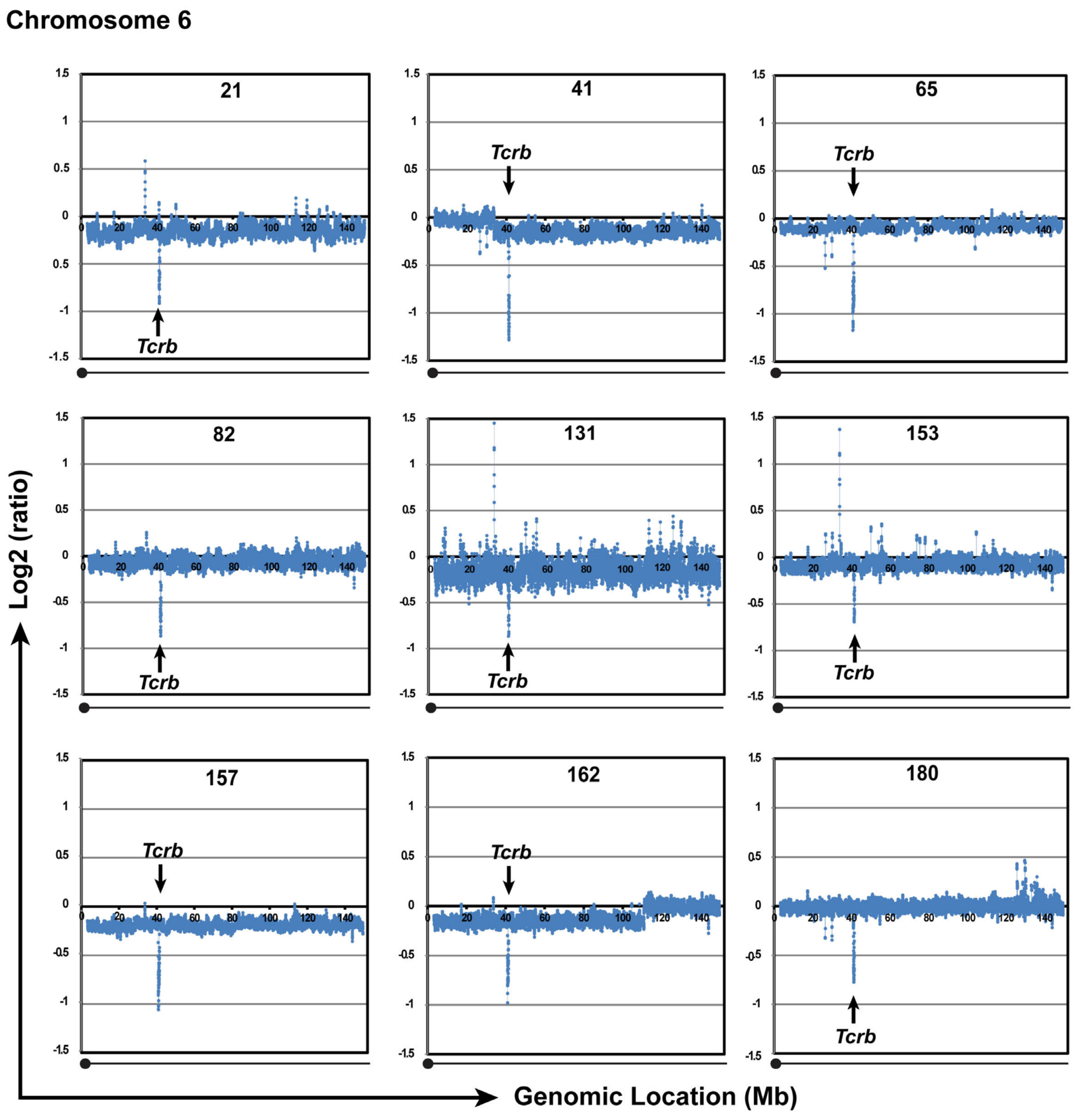

Supplementary Figure S5: Array-CGH profiles of chromosome 6 in TLs from *Trp53*+/− and *Trp53*+/− *Nucks1*+/− mice. Among all *Trp53*+/− mice tested, only TL-21 showed extreme loss at *Tcrb*. In contrast, *Tcrb* showed extreme loss in most TLs from *Trp53*+/− *Nucks1*+/− mice (TL-41, TL-65, TL-82, TL-131, TL-153, TL-157, TL-162, and TL-180). Genomic location of *Trcb* is indicated by arrow. Acrocentric chromosomes are depicted schematically under each panel.

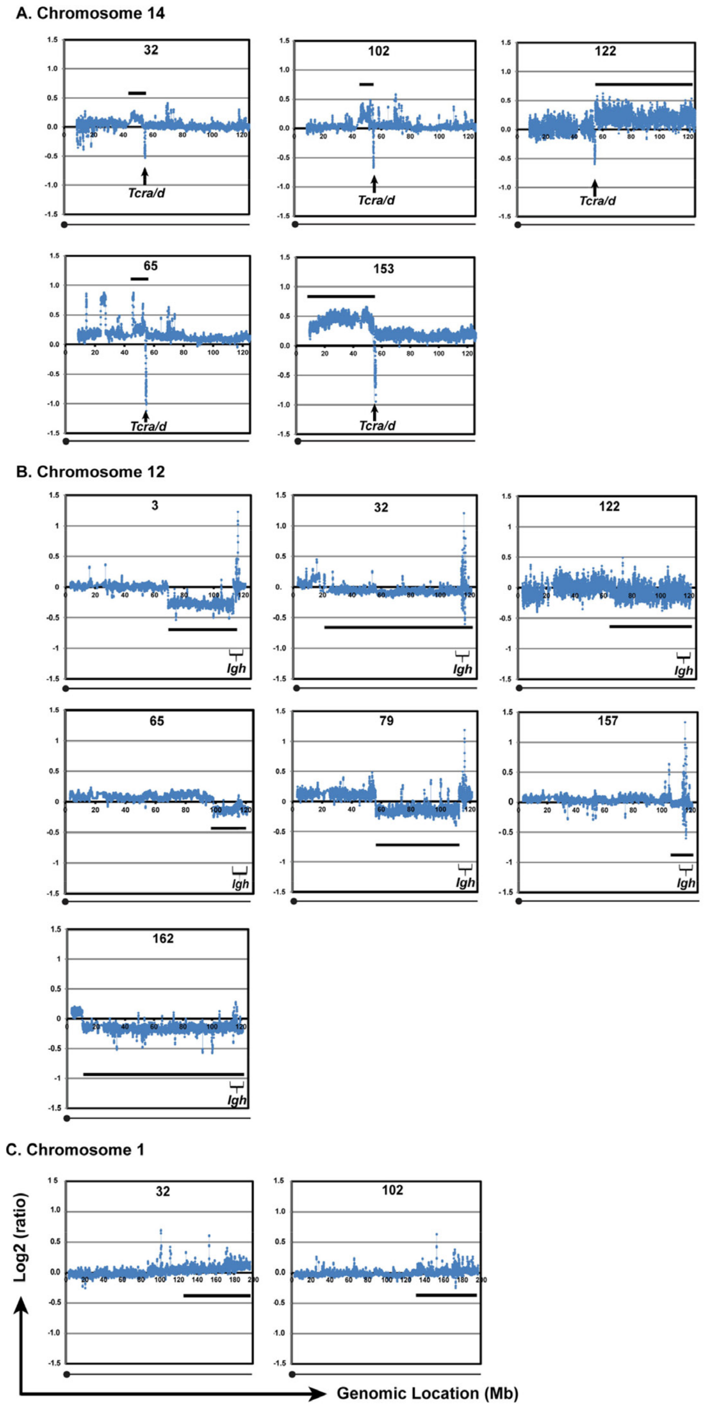

Supplementary Figure S6: Array-CGH profiles of chromosomes 14, 12 and 1 in TLs from *Trp53*+/− and *Trp53*+/− *Nucks1*+/− mice. A. Representative array-CGH profiles obtained for chromosome 14 showing gain centromeric (TL-32 and TL-102) and telomeric (TL-122) to *Tcra/d* in TLs from *Trp53*+/− mice, and gain centromeric to *Tcra/d* in TLs from *Trp53*+/− *Nucks1*+/− mice (TL-65 and TL-153). *Tcra/d* location: as indicated by arrow. Horizontal black lines: regions of gain. **B.** Representative array-CGH profiles obtained for chromosome 12 showing genomic loss in the telomeric region near *Igh* in TLs from *Trp53*+/− mice (TL-3, TL-32 and TL-122) and in TLs from *Trp53*+/− *Nucks1*+/− mice (TL-65, TL-79, TL-157 and TL-162). Horizontal black lines indicate regions of loss. **C.** Gains in the telomeric arm of chromosome 1 were observed in TLs from *Trp53*+/− mice only (here: TL-32 and TL-102), but not in TLs from *Trp53*+/− *Nucks1*+/− mice (data not shown). Horizontal black lines indicate regions of gain. Acrocentric chromosomes are depicted schematically under each panel.

Supplementary Table S1: PCR primers used

		**Oligos (5′- 3′)**
**Genotyping primers**	*Nucks1* a	AACTCAGCAAGCAGGTAGACTCTGAG
	Nucks1 b	CCAGTGATCACTTTCCAGCAGCTATTGACTG
	Nucks1 c	CAAAGGGAAAGGGTAAAGTGGTAGG
	Trp53 F1	GTGGTGGTACCTTATGAGCC
	Trp53 R1	ATAGGTCGGCGGTTCAT
	Trp53 R2	CATCGCCTTCTATCGCCTTC
**V(D)J recombination primers**	P1	ACCTATGGGAGGGTCCTTTTTTGTATAAAG
	P2	AAGACTCCTAGACTGCAGACTCAG
	P3	CAGCCCCTCTCAGTCAGACAAACC
	P4	CCCGGAGATTCCCTAACCCTGGTC
**Notch1 Type 1 deletion primers**	Type 1d_F	ATGGTGGAATGCCTACTTTGTA
	Type 1d_R	CGTTTGGGTAGAAGAGATGCTTTAC

Supplementary Table S2: Target organs of infiltrative[1] TL cells from TLs of mice with different genotypes

Genotype	Generation		Frequency (%)	Organs infiltrated
	F2	F6		
Trp53+/−	1/10	1/10	10	liver
Trp53+/−* *Nucks1+/−	4/14	4/13	29.6	liver, kidney, spleen

[1]The liver, kidney and spleen and lung were examined for secondary tumors in mice bearing TLs. Infiltration was assessed further by H&E staining and IHC of FFPE thymus tissue (anti-TdT and anti-CD3 IHC to confirm TL origin (see Figure 8 of the main manuscript)).
